# Danlou Tablet Activates Autophagy of Vascular Adventitial Fibroblasts Through PI3K/Akt/mTOR to Protect Cells From Damage Caused by Atherosclerosis

**DOI:** 10.3389/fphar.2021.730525

**Published:** 2021-11-18

**Authors:** Li Wang, Tong Wu, Chunying Si, He Wang, Ke Yue, Shasha Shang, Xiaohui Li, Yushan Chen, Huaimin Guan

**Affiliations:** ^1^ Department of Cardiovascular Medicine, The First Affiliated Hospital of Henan University of Chinese Medicine, Zhengzhou, China; ^2^ Department of Cardiovascular Medicine, The First Affiliated Hospital of Guangzhou University of Chinese Medicine, Guangzhou, China; ^3^ The First Clinical Medical College, Henan University of Chinese Medicine, Zhengzhou, China

**Keywords:** atherosclerosis, danlou tablet, autophagy, PI3K/AKT/mTOR, Chinese patent medicine

## Abstract

Danlou tablet (DLT), a commercial Chinese patent medicine, has been widely used to treat cardiovascular diseases for many years. Atherosclerosis (AS) is the leading cause of cardiovascular disease. Increasing evidence indicates that autophagy plays a vital role in the development of AS. Here we investigated whether DLT could activate autophagy to improve AS and further clarified its underlying mechanisms. In an ApoE^−/−^ mice model, the results of Oil red O, Masson’s trichrome, and H&E staining techniques showed that DLT significantly inhibited lipid accumulation and fibrosis formation in atherosclerotic plaque tissue. DLT also inhibited serum triglyceride, cholesterol, and low-density lipoprotein levels and suppressed serum levels of inflammatory factors interleukin-6 and tumor necrosis factor-α in ApoE^−/−^ mice. Moreover, DLT suppressed proliferation, migration, and invasion of human vascular adventitial fibroblasts (HVAFs) by inhibiting the PI3K/Akt/mTOR pathway. In addition, western blot analysis showed that Danlou tablet treatment decreased the expression of p62 and increased Beclin 1 and LC3 I -to-LC3 II ratios in HVAFs. The role of autophagy in treating atherosclerosis by DLT is confirmed by 3-methyladenine (autophagy inhibitor) and rapamycin (autophagy activator) in HVAFs. In summary, DLT activated PI3K/Akt/mTOR-mediated autophagy of vascular adventitial fibroblasts to protect cells from damage caused by atherosclerosis.

## Introduction

Atherosclerosis (AS), characterized by the accumulation of lipids and inflammatory cells in arterial walls, is a common pathological basis for many cardiovascular diseases (CVDs) (Falk, 2006). Although hypolipidemic agents, interventional therapy, and other conventional treatments have been used to treat this condition, atherosclerosis and its associated CVDs remain the leading cause of death worldwide (Wu et al., 2020). Therefore, it is necessary to develop novel treatment strategies for AS.

Abnormal lipid metabolism usually occurs in the initial stages of AS (Schaftenaar et al., 2016), especially concerning oxidized low-density lipoproteins (Ox-LDL), leading to the accumulation of foam cells and the formation of lipid plaques on blood vessel walls; this leads to luminal stenosis and alterations in the structure of blood vessel walls (Ference, 2018; Pirillo et al., 2018). At present, studies on the mechanism of AS mainly focus on the inflammatory response (Tedgui and Mallat, 2006; Fatkhullina et al., 2016; Zhu et al., 2018), oxidative stress (Khosravi et al., 2019), and autophagy (Grootaert et al., 2018; Tang et al., 2018). Inflammatory factors IL-6 and TNF-α play an essential role in all the stages of atherosclerosis, including plaque formation, progression, and rupture. Therefore, anti-inflammatory therapy can be a part of anti-atherosclerosis treatment (Poznyak et al., 2021). Autophagy is a biological process in which macromolecular substances and organelles in the cytoplasm are degraded in autolysosomes, meeting the metabolic needs of cells and renewing some organelles (Jenzer and Legouis, 2017; Ravanan et al., 2017). Previous studies have shown that autophagy might be a potential therapeutic strategy against AS. mTOR pathway plays a vital role in autophagy (Wang and Zhang, 2019). PI3K/Akt and MAPK signaling pathways activate mTOR pathways to inhibit autophagy, while AMPK and P53 signaling pathways suppress the activation of mTOR pathways to promote autophagy (Kim et al., 2017; Ba et al., 2019). It has been clinically verified that mTOR inhibitors, such as rapamycin, can efficiently inhibit the growth of atherosclerotic plaques (Martinet et al., 2014; Zhai et al., 2014).

Danlou tablet (DLT), a commercial Chinese patent medicine, has been approved by the China Food and Drug Administration (No. Z20050244) and is composed of 10 herbs, including *Trichosanthes kirilowii* Maxim. (Cucurbitaceae; Trichosanthis pericarpium), *Allium macrostemon* Bunge (*Amaryllidaceae*; Allii macrostemonis bulbus), *Pueraria montana* var. lobata (Willd.) Maesen and S.M.Almeida ex Sanjappa & Predeep (Fabaceae; Puerariae lobatae radix), Conioselinum anthriscoides “Chuanxiong” (Apiaceae; Chuanxiong rhizoma), Salvia miltiorrhiza Bunge (Lamiaceae; S*alviae miltiorrhizae* radix et rhizoma], *Paeonia lactiflora* Pall. (Paeoniaceae; Paeoniae radix rubra), Alisma plantago-aquatica subsp. orientale (Sam.) Sam. (Alismataceae; Alismatis rhizome), *Astragalus* mongholicus Bunge (Fabaceae; *Astragali radix*), *Davallia trichomanoides* Blume (Polypodiaceae) and *Curcuma aromatica* Salisb. (Zingiberaceae; Curcumae radix) (Gao et al., 2020). Studies have shown that DLT effectively alleviates the symptoms of angina pectoris, reduces the total cholesterol (TC) and low-density lipoprotein (LDL), mitigates inflammation, improves heart function, and reduces the incidence of cardiovascular events (Mao et al., 2016; Chen et al., 2017). Some clinical studies have shown that DLT combined with rosuvastatin dramatically reduces blood lipid levels and suppresses the formation of AS plaques in the carotid artery (Cao et al., 2015b; Liu et al., 2019). A study confirmed the role of DLT in regulating the expression of PI3K and Akt protein in an animal model of AS (Cao et al., 2015a). However, further research is still required to determine whether DLT can regulate PI3K/Akt/mTOR-mediated autophagy to alleviate AS.

This study aimed to explore the role of DLT in the development of AS and clarified its regulatory mechanisms *in vitro* and *in vivo* to provide a theoretical and practical basis for the therapeutic efficacy of DLT for AS.

## Methods

### Drug Preparation

DLT was manufactured by Jilin Connell Pharmaceutical Co. Ltd., China, following the Chinese Pharmacopoeia 2015 (Commission of Chinese Pharmacopoeia, 2015). Conioselinum anthriscoides “Chuanxiong” (52 g), *Curcuma aromatica* Salisb (52 g) and Alisma plantago-aquatica subsp. orientale (Sam.) Sam. (138 g) are ground into fine powders, then sieved and mixed. *Paeonia lactiflora* Pall (52 g), *Trichosanthes kirilowii* Maxim (86 g), and Allium macrostemon Bunge (40 g) are extracted twice by heat reflux with 70% ethanol for 1.5 h. The ethanol extract is collected and filtered, then condensed in vacuo to a final relative density of 1.25 and 1.30 (65°C). Pueraria *montana* var. lobata (Willd.) Maesen and S.M. Almeida ex Sanjappa and Predeep (138 g) and Salvia miltiorrhiza Bunge (138 g) (individually packed) are extracted three times with ethanol by heat reflux for 1 h. Extracts are collected and filtered, then condensed in vacuo to final relative densities between 1.25 and 1.30 (65°C). *Astragalus* mongholicus Bunge (114 g), *Davallia* trichomanoides Blume (26 g), and the extracted residue from Salvia miltiorrhiza Bunge are extracted twice with water for 1.5 h. Extracts are collected and filtered, then condensed in vacuo to final relative densities between 1.25 and 1.30 (65°C). The condensed extracts are mixed with the fine powder, desiccated in vacuo, ground, and pelletised. The mixture is made into 1,000 tablets (0.3 g per tablet) and film-coated. DLT-containing serum was added to the medium for the treatment of cells in culture. DLT-containing serum was prepared as follows: Twenty healthy male SD rats (Guangdong Medical Laboratory Animal Center) were randomly divided into two groups: Rats in the DLT group were administered the drug, 1,400 mg/kg/d. Normal saline was administered to rats in the blank-controlled group. Volumes administered were the same for both groups of animals. Drug or saline was given by gavage for 7 days. Rats were sacrificed 2 h after the last oral administration. Blood samples were collected from the abdominal aorta, centrifuged at 4°C, 3,000 rpm for 20 min. Supernatants were filtered, sterilized, and stored at −80°C for later use.

### Animal Model

Six-week-old male C57BL/6 mice and ApoE^−/−^ mice were purchased from Guangdong Medical Laboratory Animal Center. DLT was purchased from Jilin Cornell Pharmaceutical Co., LTD (Jilin, China). The mice were housed in plastic cages and fed with food and water at an ambient temperature of 23 ± 2°C. All of the mice were divided into six groups (*n* = 8) randomly after a week: (1) normal control group (NC), (2) atherosclerosis model group (AS model), (3) atherosclerosis model group with low-Danlou tablet (AS model + Low-DLT), (4) atherosclerosis model group with medium-Danlou tablet (AS model + Med-DLT), (5) atherosclerosis model group with high-Danlou tablet (AS model + High-DLT), and (6) atherosclerosis model group with rosuvastatin calcium tablet (AS model + RST). C57BL/6 mice were used as a normal group and fed with a normal diet. ApoE^−/−^ mice were used to establish an atherosclerosis model, fed with a high-fat diet and different doses of drug: Low-DLT (700 mg/kg/d); Med-DLT (1,400 mg/kg/d); High-DLT (2,800 mg/kg/d); rosuvastatin calcium tablet (10 mg/kg/d), via intragastric gavage, for 10 weeks. DLT powder was suspended and diluted with physiological saline and then administered to mice. After the last drug administration, the mice were euthanized by inhaling an overdose of ether; then, blood and aortic sinus samples were collected for testing. All the animal procedures were approved by the Ethics Committee of The First Affiliated Hospital of Henan University of Chinese Medicine (YFYDW2019-041).

### Histological Examination

The mice were euthanized, and the sections of aortic sinuses were harvested. The tissues were immediately fixed in 4% paraformaldehyde. Paraffin-embedded tissues were sectioned (4 µm in thickness), placed on poly-l-lysine-coated slides, and incubated for 1.5 h at 60°C. Conventional hematoxylin and eosin staining was performed (H&E), and the degree of inflammation was graded. The fibrosis level was evaluated by Masson staining of collagen accumulation according to the manufacturer’s protocol (Solarbio, China). Oil red O staining was performed using the lipid staining kit (Oil Red O) according to the manufacturer’s instructions. The stained sections were observed under a light microscope (Nikon, Ci-E).

### ELISA

Serum was harvested from the peripheral blood of the mice. The HDL level was assessed using ELISA kit (Elabscience, E-EL-M1402c) following the manufacturer’s instructions. The TC and LDL levels were assessed using the total cholesterol colorimetric test kit (Elabscience, E-BC-K109-M; NJJCBIO A113-1-1). The TG level was assessed by the triglyceride test kit (NJJCBIO, A110-1-1), according to the manufacturer’s instructions. A commercially available ELISA kit was used to measure the serum levels of inflammatory markers: IL-6 (MEIMIAN, MM-0163M1) and TNF-α (MEIMIAN, MM-0132M2).

### Cell Culture

Human vascular adventitial fibroblasts (HVAFs) were obtained from WUHAN PROCELL LIFE SCI&TECH CO., LTD. HVAFs were inoculated into a Petri dish pre-coated with polylysine and incubated at 37°C under 5% CO_2_. The culture medium was changed 48 h later for the first time and then every 3 days. The cells were then overgrown for use. OX-LDL was used to induce an atherosclerotic cell model.

### Cell Viability Assays

Cell proliferation was determined by the cell counting kit 8 assay (CCK8, Dojindo) and EdU staining assay (RIBOBIO, Cat. No. C10327) following the manufacturer’s protocol. Different groups of cells were treated as indicated in the figure legend for 3 days. At different time intervals (0, 1, 2, and 3 days) after plating, CCK-8 and EdU solutions were added to each well. CCK-8 assay was measured at 450 nm using a microplate reader (SpectraMax M3, Molecular Devices). The cells in the EdU assay were fixed in 4% paraformaldehyde overnight at room temperature and photographed under an inverted fluorescence microscope (Leica, Germany).

### Wound Healing Assays

After being seeded in 6-well plates at a confluence of about 80–90% after 24 h of incubation, the cells were wounded using a 200-µL pipette tip to scratch the monolayer of the subconfluent cell. Then, the cells underwent different drug treatments for 24 h. For the next 48 h, the cells were cultured in a serum-free medium and allowed to migrate. The images of cell migration were captured using an inverted microscope. The migration speed was calculated by dividing the length of the gap by the wound areas in the captured images.

### Transwell Assays

Millicell chambers with polycarbonate microporous membranes and artificial matrigel were used. The cells were digested, collected, and washed with phosphate buffered saline (PBS) once and suspended with a serum-free medium to adjust the concentration to 2×10^5^/ml; 800 μL of 10% serum medium was added to the bottom chamber, and then 100–150 μL of cell suspension was added to the upper chamber. The culture continued for 12 h in the incubator. The chamber was retrieved and fixed with methanol for 30 min at room temperature. Next, the chambers were moved to a well with 800 μL of crystal violet solution to be stained at room temperature for 15 min. The cells on the membrane surface at the bottom of the upper chamber were carefully wiped off using a wet cotton swab. Then the membrane was carefully removed with forceps and dried upward. The membrane was transferred to a slide and sealed with neutral resin, and photographs were taken under an inverted fluorescence microscope. Nine random fields were selected and counted.

### Immunofluorescence Microscopy Analysis

The cell slides were fixed with 4% paraformaldehyde solution at room temperature. After 15 min, the cells were washed with PBS three times and permeabilized by immersion in 0.3% Triton X-100 in PBS for 10 min. Then the slides were blocked with 2% bovine serum albumin (BSA) in PBS for 1 h at room temperature and incubated with the primary antibodies Vimentin (ab8978, 1:500, Abcam) and LC3B (ab229327, 1:200, Abcam) at 4°C overnight. Afterward, the slides were stained with fluorescently-labeled secondary antibody for 1 h at room temperature and mounted by VECTASHIED solution (vector) with DAPI. The images were taken under a confocal fluorescence microscope (Olympus, Japan) and analyzed by NIS elements imaging software.

### Western Blot

The total protein extraction was performed with RIPA lysis buffer (mixed with phosphatase and protease inhibitor), and protein quantification was carried out with a BCA kit. The samples were separated on 4–20% SDS-PAGE gel and then transferred to a nitrocellulose membrane. After blocking by 5% skimmed milk for 1 h, the membrane was incubated overnight at 4°C with specific antibodies: p-mTOR (#5536, 1:1,000, CST), mTOR (#2983, 1:1,000, CST), AKT (#4691, 1:1,000, CST), p-AKT (#4060, 1:1,000, CST) p-S6 (#2215, 1:1,000, CST), LC3A/B (#12741, 1:1,000, CST), P62 (#5114, 1:1,000, CST), Beclin-1 (ab210498, 1:1,000, Abcam), ICAM-1 (ab53013, 1:1,000, Abcam), VCAM-1 (ab115135, 1:1,000, Abcam), and GAPDH (ab8245, 1:5,000, Abcam). After being washed with TBST buffer three times, the membrane was further incubated with the secondary antibody for 1 h, washed three times with TBST buffer, and then exposed in ECL developer in Image lab.

### Transmission Electron Microscope Analysis

The samples were dropped on a carbon support membrane copper mesh for 3–5 min, and then a filter paper was used to absorb excess liquid. Then, 2% phosphotungstate was dropped on the carbon support membrane copper mesh for 2–3 min and air-dried at room temperature. The images were observed under a transmission electron microscope (HT7700 HITACHI) and captured for analysis.

### Statistical Analysis

GraphPad Prism software was used to perform statistical analyses. The experimental data were expressed as the mean ± SD, analyzed by one-way ANOVA, followed by Student’s t-tests. Differences with *p* < 0.05 were considered statistically significant (*p < 0.05, **p < 0.01, and ***p < 0.001).

## Results

### Danlou Tablet Relieves Pathological Lesion of AS in ApoE^−/-^ Mice Model

**FIGURE 1:**
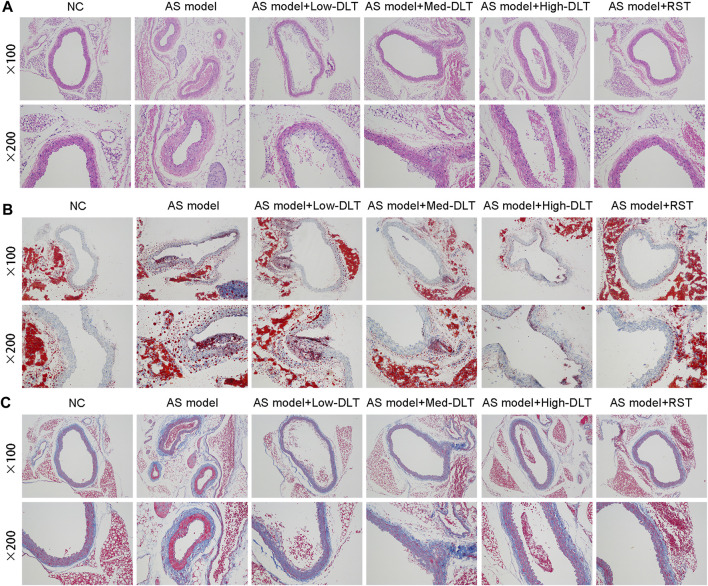
Danlou tablets relieve the pathological lesion of AS in the ApoE^−/−^ mice model. **(A)** Representative photomicrographs of hematoxylin eosin-stained aortic sinuses. **(B)** Representative photomicrographs of Oil red O-stained aortic sinuses. **(C)** Masson trichrome staining to show the fibrosis in different groups.

To detect the influence of the medical tablet on AS *in vivo*, we first established an AS mouse model using the ApoE^−/−^ mice by a high-fat diet, and significant AS plaque was observed as previously described (Emini Veseli et al., 2017). To further elucidate the therapeutic effect of Danlou tablet, different doses of Danlou tablet and rosuvastatin calcium tablet were administrated, and AS plaque tissues were harvested from the mice, followed by the assessment for the degree of the lesion. Histological examinations demonstrated thicker plaque in ApoE^−/−^ mice than the control mice. While Danlou tablets significantly reduced the degree of pathological lesions in a dose-dependent manner based on H&E staining (Figure 1A), they exhibited efficacy comparable to traditional rosuvastatin calcium tablets. In addition, lipid accumulation and fibrosis formation were alleviated in the Danlou tablet-treated ApoE^−/−^ mice according to the Oil Red O and Masson trichrome staining, red lipid droplets accumulated mostly at the edge of AS lesions (Figures 1B,C).

**FIGURE 2:**
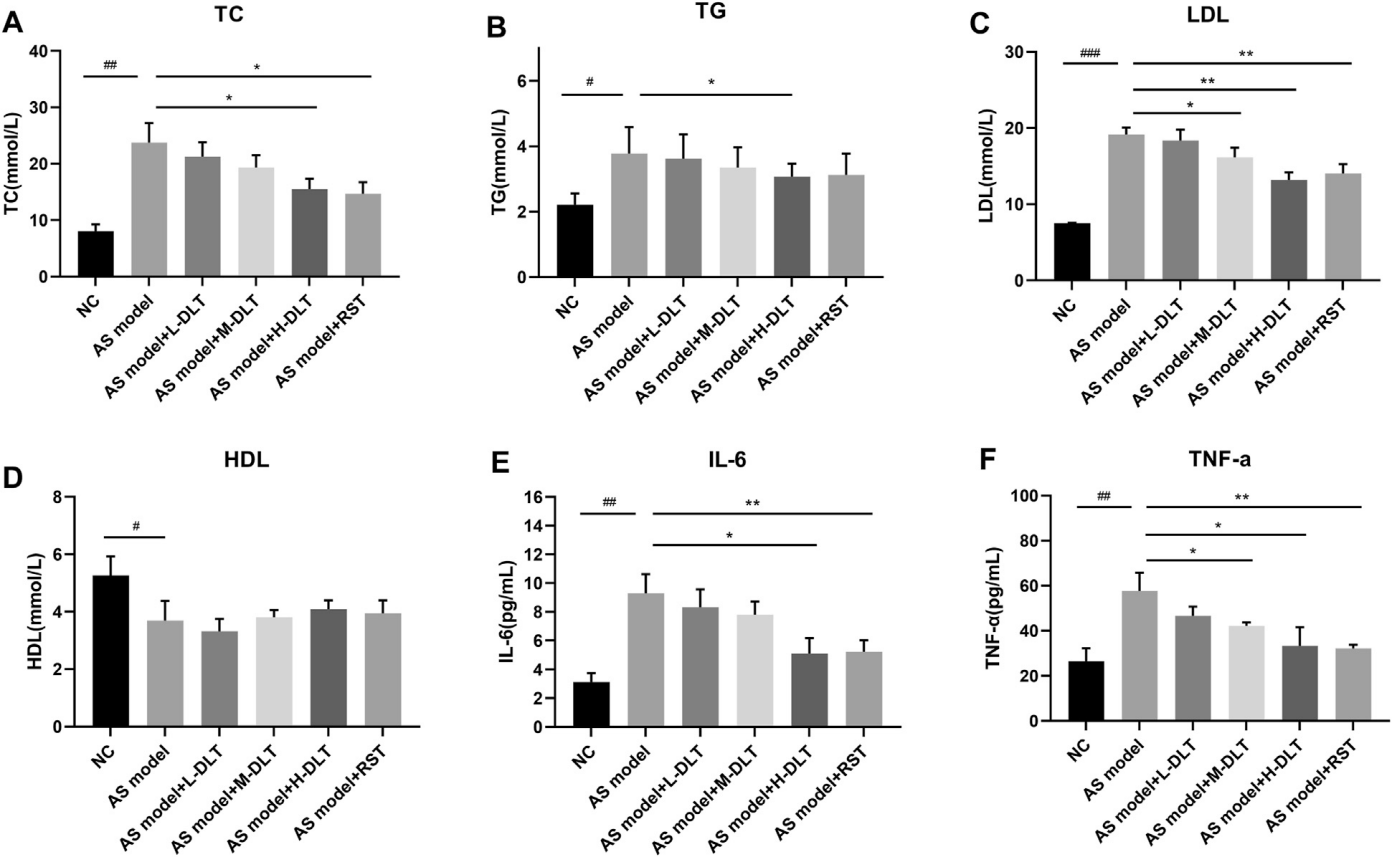
The AS-related cytokines detection in a Danlou tablet-treated ApoE^−/−^ mice model. **(A–D)** Lipid levels were detected by ELISA and biochemical test, including LDL, HDL, TC, TG. **(E, F)** ELISA kits were used to check the immune factors (IL-6 and TNF-α) in different groups (#*p* < 0.05, ##*p* < 0.01, ###*p* < 0.001 AS model group vs. normal control group; *p < 0.05, **p < 0.01, ***p < 0.001 drug treatment group vs. AS model group, *n* = 8, tested by one-way ANOVA).

In order to investigate the biochemical basis that leads to the pathological results, we collected serum samples from the mice to determine the blood total cholesterol (TC), triglyceride (TG), high-density lipoprotein (HDL), and low-density lipoprotein (LDL) levels and inflammatory factors, like IL-6 and TNF-α. Interestingly, Danlou tablets significantly reduced TC (Figure 2A), TG (Figure 2B), and LDL (Figure 2C) levels but did not alter the HDL (Figure 2D) levels in ApoE^−/−^ mice. Of note, two inflammatory factors, IL-6 (Figure 2E) and TNF-α (Figure 2F), were strongly decreased by high-dose treatment of Danlou tablets. Collectively, these data underline the capacity of Danlou tablets to alleviate AS *in vivo*.

### DLT Inhibits the Ox-LDL Effects in HVAF Cells

**FIGURE 3:**
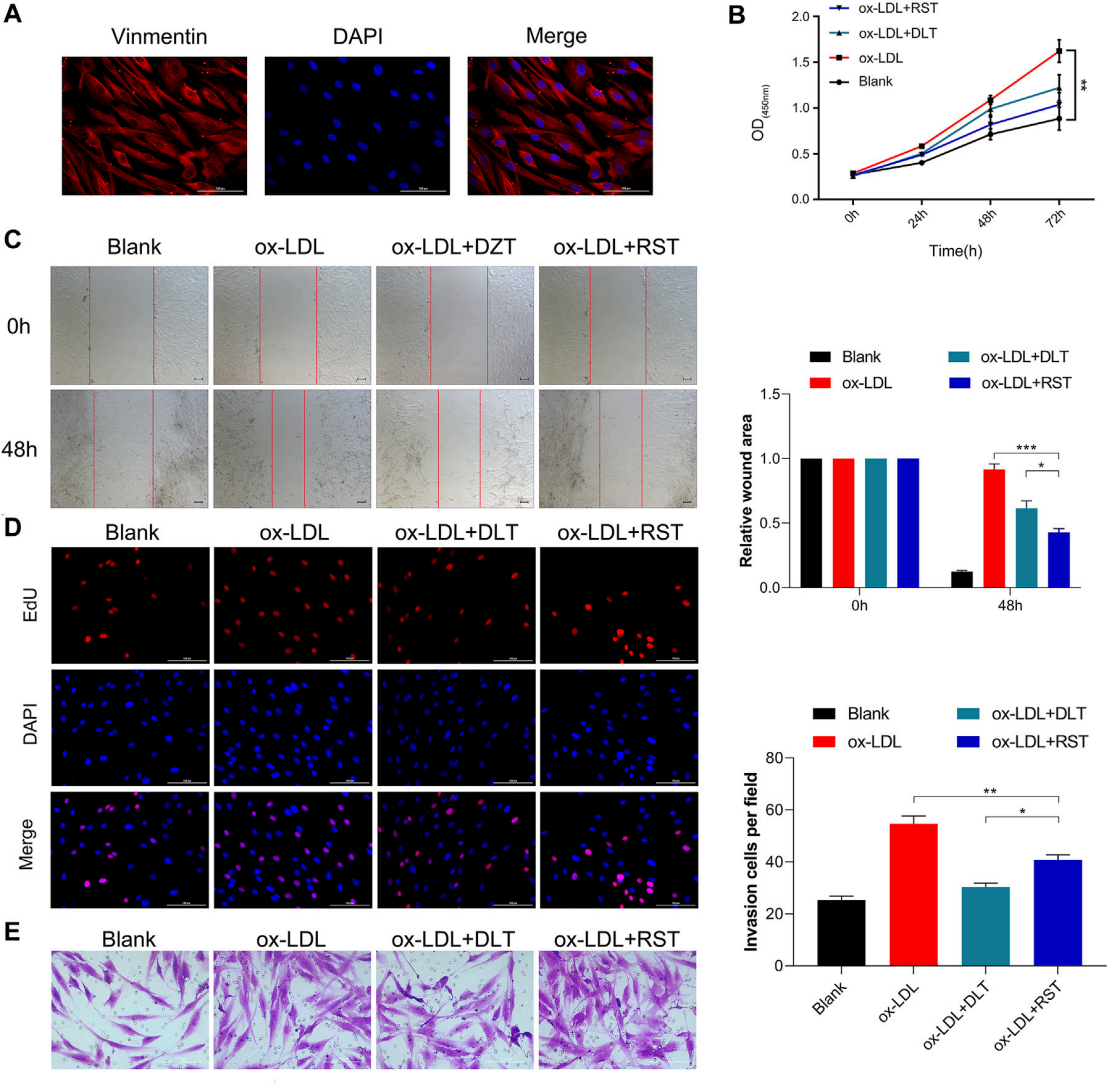
DLT inhibits the ox-LDL effects in the HVAF line. **(A)** Vimentin staining was used to identify the human vascular adventitial fibroblasts (HVAFs). **(B, D)** Cell proliferation was evaluated by CCK8 and EDU staining. **(C, E)** Wound healing and transwell assay showed the migration ability in the ox-LDL-induced model and DLT treatment group (*p < 0.05, **p < 0.01, ***p < 0.001).

**FIGURE 4:**
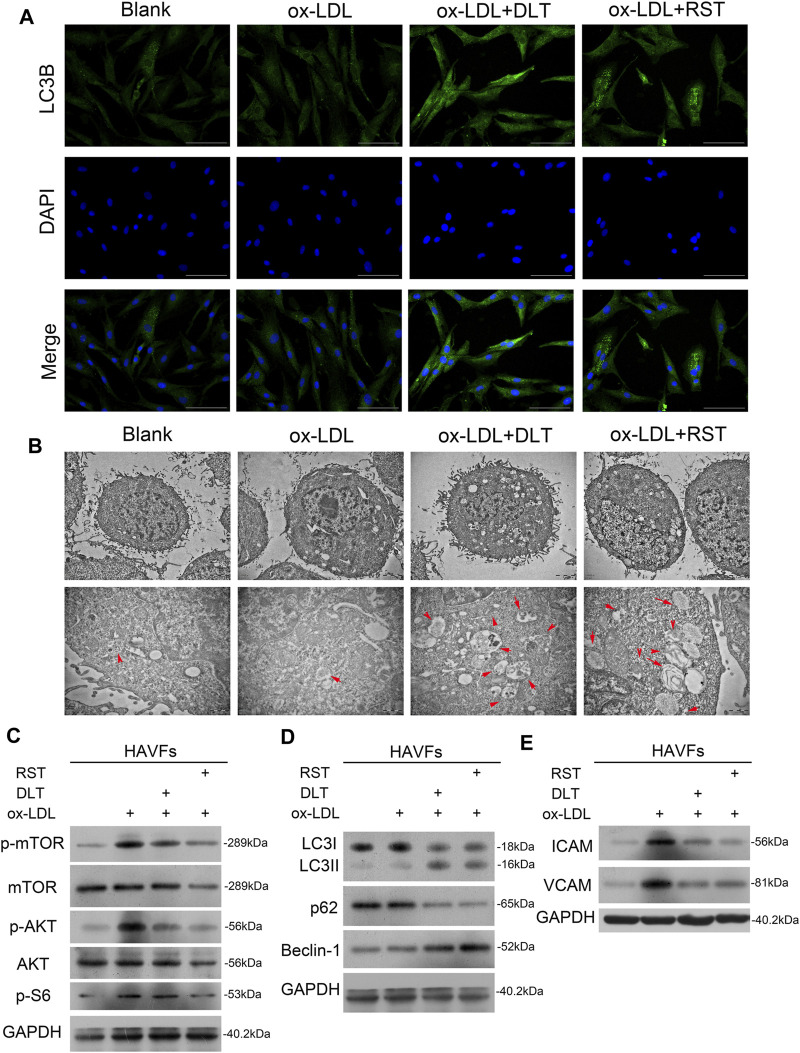
DLT activates the autophagy pathway in HVAFs through the PI3K/Akt-mTOR pathway. **(A)** Immunofluorescence staining of LC3 to show the activation of autophagy in the DLT treatment group. **(B)** Transmission electron microscopy analysis to show the autophagosome in the DLT treatment group (red arrow). **(C)** Western blot showed DLT treatment reversed the ox-LDL-induced activation of the PI3K/Akt-mTOR pathway. **(D)** The autophagy marker expression was detected by western blotting and increased in the DLT treatment model. **(E)** The HVAFs activation markers ICAM-1 and VCAM-1 were checked in the DLT-treated and ox-LDL model.

LDL could accumulate in fibroblasts at the edge of some AS lesions, which might be an early indication of atherosclerosis (Lees et al., 2001). We used a human vascular adventitial fibroblasts (HVAFs) cell line to identify the mechanism of Danlou tablets (DLT) in alleviating AS. We confirmed the high expression of vimentin, a well-known marker of HVAFs, by immunofluorescence cytometry (Figure 3A). Next, an AS cell model was established by adding ox-LDL to the HVAFs culture medium. Therefore, the effect of DLT was evaluated *in vitro* by this AS cell model. As indicated in Figure 3B, DLT-containing serum notably reduced the growth of HVAFs, which were significantly stimulated by ox-LDL through the CCK8 experiment. The anti-proliferation ability of DLT was also similar to rosuvastatin (RST), consistent with the EDU staining assay (Figure 3D). The cell wound healing assay and transwell assay illustrated that DLT significantly down-regulated cell migration and invasion ability compared to RST treatment (Figures 3C,E). After activation, HVAFs can secrete a large amount of intercellular adhesion molecule-1 (ICAM-1), vascular cell adhesion molecules (VCAM-1) (Liu et al., 2010), chemokines, and inflammatory factors to accelerate the chemotaxis of monocytes and lymphocytes and mediate the inflammatory response. Therefore, we used western blotting to detect the ICAM-1 and VCAM-1 expression after ox-LDL treatment, and the results were consistent with the phenotype of HVAFs (Figure 4E). In short, these data suggest that DLT might slow down the formation of AS by suppressing the activity of HVAFs.

### DLT Activates Autophagy in HVAFs

It is well established that vascular endothelial cells undergo autophagy to protect their structures from inflammation and oxidative stress when stimulated by ox-LDL in AS. Here, we speculated that the reduced activity of HVAFs might result from the alteration of autophagy. To directly determine the degree of autophagy in HVAFs, we detected the LC3B level by immunofluorescence cytometry. As shown in Figure 4A, DLT increased the expression of LC3B, indicating a high rate of autophagy. Moreover, we also observed the extensive formation of phagosomes under the transmission electron microscope (Figure 4B). Consistent with this finding, increased expression of Beclin1 and LC3 I-to-LC3 II ratio further supported our hypothesis (Figure 4D). In addition, the increased expression of p62 in the autophagosome degradation stage is usually regarded as a sign of inhibited autophagy activity. We also showed *via* Western blot that DLT reduced the expression of p62 (Figure 4D). Altogether, we concluded that DLT activates autophagy to protect HVAFs from damage caused by AS.

### DLT Activates Autophagy Through PI3K/Akt/mTOR Pathway

To explain our findings, we analyzed the signal molecular mechanisms involved in autophagy as previous studies have demonstrated that the mTOR pathway is a key link of the autophagy process. PI3K/Akt and MAPK signaling pathways induce the activation of mTOR to inhibit autophagy. For instance, the activated PI3K-I generates PIP3 in cells, activating Akt with the assistance of PDK1, inhibiting the TSC1/2 complex and activating mTORC1 (Dieterle et al., 2014). Ribosomal protein S6 is a downstream target of mTOR. Levels of phospho-S6 are a frequently used marker of activation of the mTOR pathway. Phosphorylation of S6 exerts an inhibitory effect on autophagic proteolysis (Hay and Sonenberg, 2004; Lu et al., 2019). To exploit the contribution of mTOR pathways to the alteration of autophagy in HVAFs, we analyzed the indicated signaling pathways by western blotting. As the level of phosphorylated mTOR, Akt, and S6 were notably decreased by the administration of DLT (Figure 4C), these findings indicate that DLT activated autophagy partly through inhibiting the mTOR via the PI3K/Akt pathway.

### mTOR Pathway Agonist Reverses the Effect of DLT

**FIGURE 5:**
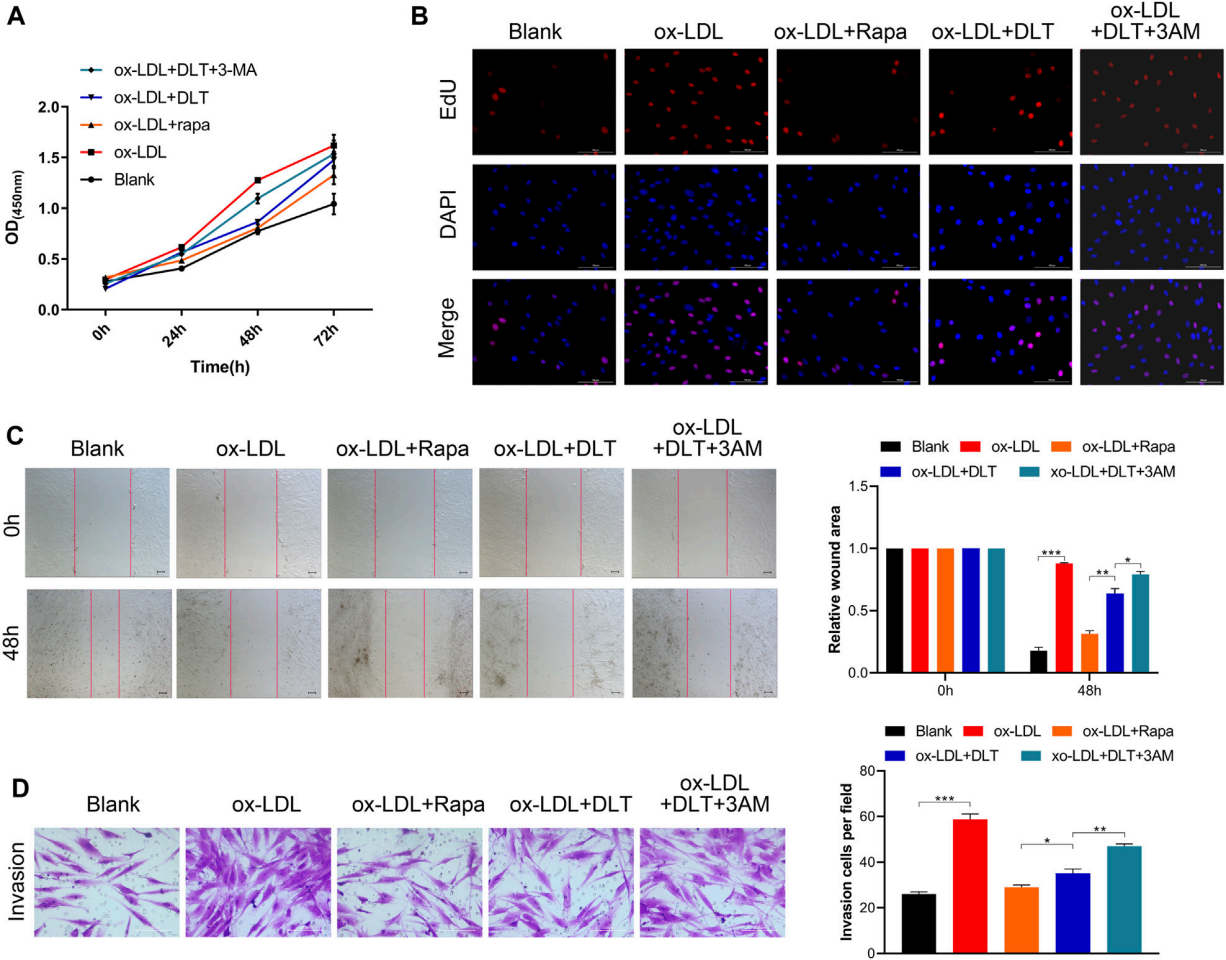
mTOR pathway agonist reverses the effect of DLT. **(A, B)** CCK8 and EDU assay were used to evaluate the proliferation of DLT and mTOR pathway agonist effects in the AS model. **(C, D)** The wound healing and transwell showed that the mTOR pathway agonist could increase the DLT-induced migration in the AS cell model (*p < 0.05, **p < 0.01, ***p < 0.001).

**FIGURE 6:**
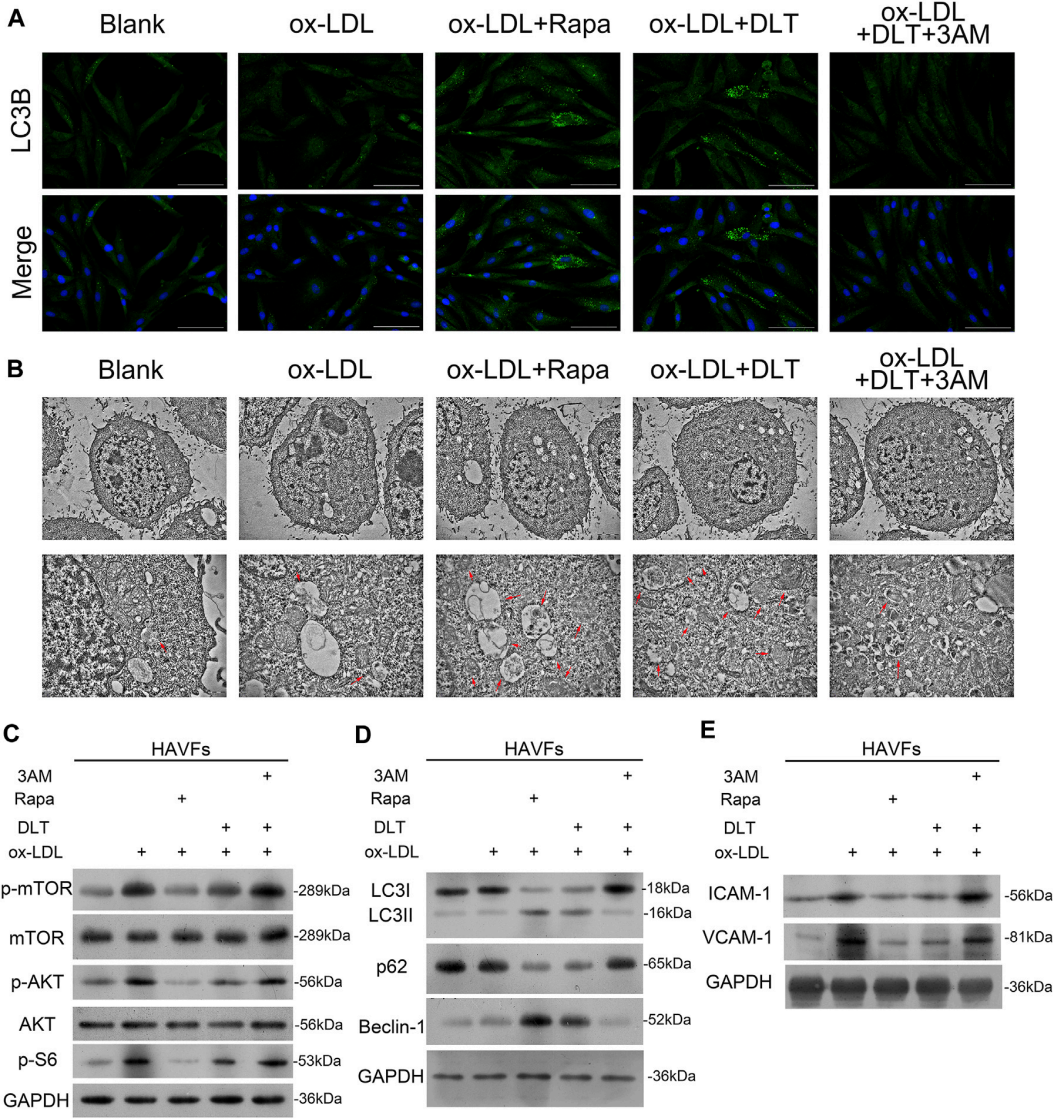
mTOR pathway agonist decreases autophagy by DLT in the AS model. **(A, B)** Immunofluorescence staining of LC3 and transmission electron microscopy analysis to show the activation of autophagy in the DLT treatment group and inhibition by the mTOR agonist. **(C)** Western blot showed the activation of the PI3K/Akt-mTOR pathway by the mTOR agonist. **(D)** The autophagy marker expression was detected by western blotting and reduced in the DLT-mTOR agonist double treatment group. **(E)** The HVAFs activation markers ICAM-1 and VCAM-1 were checked in the DLT-mTOR agonist double treatment group.

To verify the influence of DLT on the mTOR signaling pathway, we further performed 3-MA, an mTOR pathway agonist (Wu et al., 2013), to study whether activated autophagy could be reversed. As expected, the decreased proliferation of HVAFs was distinctly restored by 3-MA (Figures 5A,B). Of note, the efficiency of DLT in the cells was comparable to Rapa, a conventional antagonist of the mTOR signaling pathway (Cai et al., 2018). The results presented in Figures 5C,D also indicated that 3-MA increased the motility of HVAFs. The raised expression of ICAM-1 and VCAM-1 via western blotting (Figure 6E) further manifested the recuperative activity of HVAFs.

3-methyladenine (3-MA) is a widely used inhibitor of autophagy due to its inhibitory effect on PI3K and suppression of the conversion of LC3-I to LC3-II (Zhang et al., 2020). Here we observed reduced autophagy by the lower expression of LC3B by immunofluorescence cytometry (Figure 6A) and decreased formation of phagosomes under the transmission electron microscope in the 3-MA group (Figure 6B). The lower expression of Beclin1 and LC3 I -to-LC3 II ratio by western blotting were consistent with the phenotype. In addition, we also demonstrated increased expression of P62 via western blotting (Figure 6D). Data from western blotting also confirmed that 3-MA reversed the activated autophagy by DLT by stimulating the mTOR via the PI3K/Akt pathway (Figure 6C). Treatment with DLT decreased p-mTOR protein levels, increased conversion of LC3-I to LC3-II, and activated autophagy compared with the ox-LDL group, but the effect could be reversed by 3-MA (Figures 6 B–D). In conclusion, the therapeutic effect of DLT could be reversed by inhibiting autophagy through the mTOR pathway agonist, indicating the pivotal role of the PI3K/Akt/mTOR pathway in this process.

## Discussion

Atherosclerosis, a complex and common condition involving chronic inflammation and vascular remodeling processes, is the pathological basis of many cardiovascular diseases (Peng et al., 2016). Apolipoprotein E (ApoE) is a protein involved in the transformation and metabolism of lipoproteins. Lack of ApoE might result in the accumulation of cholesterol in the circulating blood, leading to the formation of atherosclerotic lesions (Wang et al., 2017). The pathological characteristics of ApoE^−/−^ mice with atherosclerosis are very similar to those of humans with atherosclerosis. Moreover, compared with the ApoE^−/−^ mice with normal diets, there were significantly increased atherosclerotic plaques, inflammatory cells, deposits of neutral lipid droplets, and decreased matrix fiber components in the fiber caps in ApoE^−/−^ mice fed a high-fat diet (Zhou et al., 2009). Therefore, the high-fat diet-fed ApoE^−/−^ mouse model is ideal for studying atherosclerosis. Therefore, a high-fat diet-fed ApoE^−/−^ mouse model was used in our study to explore the effect and underlying mechanism of DLT on atherosclerosis.

Danlou tablet, a traditional Chinese medicine, consists of 10 herbs and has been approved to treat ischemic heart disease for a long time (Mao et al., 2016; Ding et al., 2019). The Danlou tablet has been demonstrated to alleviate phlegm and stasis mutual obstruction syndrome, reduce serum inflammatory factor levels, and improve the quality of life in patients with unstable angina pectoris (Wang et al., 2016). Current research data indicate that Danlou tablet inhibits atherosclerosis *via* various mechanisms, including the inhibition of Angptl4 protein level through the HIF-1α-Angptl4 mRNA signaling pathway (Tang et al., 2020), inhibition of the NF-κB-mediated inflammatory response (Gao et al., 2020), and prevention of PPARα/ABCA1-regulated lipid deposition (Hao et al., 2019). In our research, Danlou tablets significantly relieved the pathological process of atherosclerosis in ApoE^−/−^ mice, including decreased lipid accumulation, fibrosis formation, and inflammation responses. Interestingly, the capability of Danlou tablets is even comparable to rosuvastatin.

Autophagy is a biological process in which cells degrade damaged proteins and disordered organelles *via* autolysosomes. It plays an essential role in maintaining cell homeostasis (Jenzer and Legouis, 2017; Ravanan et al., 2017). Growing evidence shows that impaired autophagy is responsible for atherosclerotic plaque development, disordered lipid metabolism, and vascular endothelial cell dysfunction (Peng et al., 2016). Inflammation is responsible for development of atherosclerosis (Li et al., 2017). Recently, a pivotal regulatory role has been confirmed in the ATG16L1 (autophagy-related 16-like 1)-deficient mice for autophagy in generating proinflammatory cytokines, including IL-1β and IL-18 (Saitoh et al., 2008). This evidence suggests that autophagy plays a crucial role in the regulation of inflammatory responses. Dysfunctional autophagy significantly activates the inflammatory response, promoting atherosclerosis (Razani et al., 2012). In our study, DLT significantly reduced inflammatory factor IL-6 and TNF-α levels in AS model, activated autophagy of vascular adventitial fibroblasts, which may be part of its anti-atherosclerosis mechanism. Moreover, a growing body of evidence demonstrates that mTOR inhibitors, such as rapamycin, possess obvious anti-atherosclerotic effects and should be considered supplementary therapy for atherosclerosis. Therefore, autophagy-mediated inflammatory responses might be a potential therapeutic strategy against atherosclerosis, opening up new horizons for the treatment of atherosclerosis.

In the human vascular adventitial fibroblasts cell model, we discovered that Danlou tablets prominently suppressed cell proliferation and mobility. Furthermore, Danlou tablets activated the autophagy process by regulating the PI3K/Akt/mTOR pathway to intervene in the activity of HVAFs. As was expected, the autophagy inhibitor 3-MA significantly suppressed the effect of Danlou tablets on atherosclerosis, while rapamycin, an autophagy activator, significantly enhanced the effect of Danlou tablets on atherosclerosis. This evidence suggests that Danlou tablets can activate PI3K/Akt/mTOR-mediated autophagy to improve atherosclerosis.

*In-vitro* experiment with medicated serum of traditional Chinese medicine has been applied to diseases research (Shi et al., 2016; Deng et al., 2021). In order to find the appropriate serum concentration to evaluate drug effect, the animal is usually gavaged with the equivalent of 1–10 times the clinical dose for several days, and then the medicated serums are collected 0.5–2 h after the last dose. Finally, the serums are diluted with different concentration for preliminary experiments to determine the concentration for *in vitro* experiments (Ma et al., 2017). The human dosage of DLT is 4.5 g per day (75 mg/kg/d for a 60 kg person) according to drug instructions. A guide for human and animal dose conversion indicates that drug consumption for rat is 6.2 times that for humans (Nair and Jacob, 2016). In our experiment, DLT (1,400 mg/kg/day) with 3 times the effective dosage for human body was given to rats by intragastric administration to prepare medicated serums. Then, DLT-containing serums at concentrations of 2.5, 5, 10, and 20% were used for preliminary *in vitro* experiments respectively. The results showed that the DLT-containing serum at the concentration of 10% exerted the most obvious activation effect on autophagy. Therefore, according to the results of the preliminary experiment, the DLT-containing serum at the concentration of 10% was used for *in vitro* experiments.

We have discovered part of the mechanism of DLT in the treatment of atherosclerosis through *in vivo* and *in vitro* experiment, but there are still several problems to be solved. First, only the effect of DLT on vascular adventitia fibroblasts was studied in our experiments, and its effects on vascular endothelial cells, smooth muscle cells, and immune cells remain to be further studied. Second, DLT is a mixture of multiple compounds, so it is difficult to study its core active ingredients. A total of 33 representative components in 20 batches of DLT were simultaneously quantified by ultra-high performance liquid chromatography (UHPLC), and therefore proved the stability of DLT pharmaceutical ingredient (Lin et al., 2020). The study also shows that Danshensu and Lithospermate B are the most important quantitative markers for DLT quality control, and are probably the core chemical substance basis for its pharmacological effects, which is worthy of further research. Finally, the interaction between molecular substances in other signaling pathways should be fully considered; DLT may also exert its anti-atherosclerotic effect through other mechanisms. In the future, network pharmacology can be used to predict the target of DLT’s anti-atherosclerotic effect and evaluate its other potential pharmacological effects.

## Conclusion

In conclusion, we demonstrated that Danlou tablets provoke the autophagy of vascular adventitial fibroblasts by regulating the PI3K/Akt/mTOR pathway to protect cells from damage caused by atherosclerosis. Thus, Danlou tablets should be regarded as a new candidate for atherosclerosis treatment.

## Data Availability

The raw data supporting the conclusion of this article will be made available by the authors, without undue reservation.

## Abbreviations

AS, atherosclerosis; HVAFs, human vascular adventitial fibroblasts; LDL, low-density lipoprotein; TC, total cholesterol; TG, triglyceride; DLT, danlou tablet; RST, rosuvastatin; ICAM-1, intercellular adhesion molecule-1; VCAM-1, vascular cell adhesion molecules-1.
